# ﻿Clavigeritae (Coleoptera, Staphylinidae, Pselaphinae) of the Arabian Peninsula with the description of a new species of *Corynotopsis* Jeannel, 1951 from Oman

**DOI:** 10.3897/zookeys.1198.119152

**Published:** 2024-04-24

**Authors:** Peter Hlaváč, Petr Baňař, Dominik Stočes

**Affiliations:** 1 Department of Zoology, Fisheries, Hydrobiology and Apiculture, Faculty of AgriSciences, Mendel University, Zemědělská 1, Brno, CZ-613 00, Czech Republic Mendel University Brno Czech Republic

**Keywords:** *Articerodes*, *Commatocerus*, new country records, rove beetles, taxonomy

## Abstract

*Corynotopsisomanicus***sp. nov.** of the myrmecophilous supertribe Clavigeritae is described from Oman. The type series was collected at night and the ant host species remains unknown. *Corynotopsisscotti* Jeannel, 1951 is newly recorded for Yemen, and *Commatocerusconcinnus* Besuchet & Cuccodoro, 2011 for Oman. *Lasius* Fabricius, 1804 and *Lepisiota* Santschi, 1926 are, for the first time, determined as hosts of the latter species. The problematic taxonomic status of *Corynotopsisscotti* is discussed. A distribution map of all Clavigeritae known from the Arabian Peninsula is provided.

## ﻿Introduction

The obligate myrmecophilous supertribe Clavigeritae is very poorly represented on the Arabian Peninsula. To date only three genera with three species have been recorded from this vast area. *Articerodessyriacus* Saulcy, 1865 is a widespread species, known from Greece, Iran, Iraq, Israel, Lebanon, Tadzhikistan, Turkey, Uzbekistan, Yemen (Socotra I.) and Ethiopia (Schülke and Smetana 2015). *Commatocerusconcinnus* Besuchet & Cuccodoro, 2011 was described from Ras Al Khaimah (United Arab Emirates), and *Corynotopsisscotti* Jeannel, 1951 originally described from Ethiopia, and later recorded from Saudi Arabia (Besuchet 1999), is here recorded from Yemen.

The aim of this paper is to provide a synopsis of Clavigeritae of the Arabian Peninsula with the description of a new species, *Corynotopsisomanicus* sp. nov., as well as to provide new records and the host ant association for *Commatocerusconcinnus*.

## ﻿Material and methods

Specimens prepared for the morphological study were examined with a Leica S8APO stereoscopic microscope with diffuse lighting at magnifications up to 128×. Habitus images were taken with a Canon EOS 6D in combination with a Canon MP-E65 1–5× macro lens; final images were composed from partial photographs using Helicon Focus 7.0. and post-processed in Adobe Photoshop 2020.

The aedeagi were studied using a Zeiss transmitted-light microscope at magnifications up to ×500. They were dissected and preserved in Euparal on plastic label pinned together with the specimen. All drawings were made using a drawing tube.

The head length was measured from the occipital constriction to the anterior margin of the frontal rostrum; head width was measured across the eyes; elytral length was measured along the suture; width means maximum width of pronotum, elytra, etc. The body length is a combined length of the head, pronotum, elytra, and abdomen. The length of basal and apical parts of the median lobe were measured in dorsal view.

The terminology applied here follows Chandler (2001), except we use ‘ventrite’ instead of ‘sternite’ when discussing ventral thoracic structures. Paired structures are treated as singular. The description is for males; the differences for females are treated in the sexual dimorphism section.

Label data are cited verbatim, with slashes (/) separating lines of text. The comments of the authors are in square brackets. All labels of the studied material are printed. All type specimens were provided with the following red printed label: HOLOTYPE or PARATYPE, *Corynotopsisomanicus* sp. nov., P. Hlaváč det., 2023.

Specimens studied herein are deposited at the following institutes and collections:

**MHNG**Muséum d’histoire naturelle, Geneva, Switzerland

**MMBC**Moravian Museum, Brno, Czech Republic

**NMPC**National Museum (Natural History), Prague, Czech Republic

**PCJP** private collection of Jan Pelikán, Hradec Králové, Czech Republic.

**PCJV** private collection of Jaroslav Větrovec, Hradec Králové, Czech Republic.

**PCPH** private collection of Peter Hlaváč, Prague, Czech Republic.

**PCTK** private collection of Tomáš Kopecký, Hradec Králové, Czech Republic.

## ﻿Taxonomy

### ﻿ Corynotopsis omanicus sp. nov.

Taxon classificationAnimaliaColeopteraStaphylinidae

55408CAC-B180-5574-8C9E-C6B811FE603B

https://zoobank.org/661F1795-811C-4B6B-8535-1E33032B6D12

Figs 1A , 2A, B

#### Type material.

***Holotype***, ♂: **Oman**: one label “OMAN, DHOFAR PR. / 18 km NW of Sadah / near Lagga Shalyon / 422 m / 17°11'10.046"N, 54°56'34.295"E / Větrovec J. leg. 20.9.2022“ [white, printed] (NMPC). ***Paratypes*** (8 ♂♂, 2 ♀♀): 2 ♂♂: same data as for holotype. (PCJV, PCPH). 1 ♂: one label “OMAN – DHOFAR PR. / E of Aytin, Wadi Hinnah / wadi - Baobab Forest 300 m / 17°3'12.964"N, 54°36'32.143"E / Větrovec J. leg. 21.9.2022” [white, printed] (PCJV). 1 ♀: one label “Oman, 21.9.2022, Dhofar / Govern. E of Aytin, Wadi / Hinnah, wadi - Baobab Forest / 17.0536011°N, 54.6089286°E, / Lgt. T. Kopecký, 300 m” [white, printed] (PCTK). 5 ♂♂, 1 ♀: two labels “S Oman, Dhofar Gov. / Sadah, 18 km NW / 20.9.2022 / lgt. Jan Pelikan” [white, printed], “near Lagga Shalyon / 17°11'10.046"N, 54°56'34.295"Е wadi / at UV light“ [white, printed] (PCJP, PCPH, NMPC, MMBC).

#### Diagnosis.

Head lacking frontal and vertexal foveae; clypeus massive, well-visible on sides as well as in front of rostrum; eyes prominent; disc of venter part of head smooth, sides with rugose surface, posterior tentorial pits separated; antennae hexamerous; antennomere 3 and 4 subequal; terminal antennomere longest, cylindrical, about five times as long as 5 and three times as long as wide; pronotum lacking antebasal median foveae, with weakly-defined lateral fovea; lacking sulci; elytra lacking basal foveae, with short discal and sutural striae; lateral posterior margin with weak, short trichome; abdomen with basal basin of composite tergite transverse, almost entire but with two small protrusions forming two, small lateral lodges; first visible sternite (III) about half as long as second (IV), sternites (IV–VII) with median impression; legs stout; mesofemur with basal bifurcate thorn; mesotibia with predistal spine; aedeagus about 2.20 times as long as wide; dorsal circular diaphragm well-defined.

#### Description.

**Figure 1.**
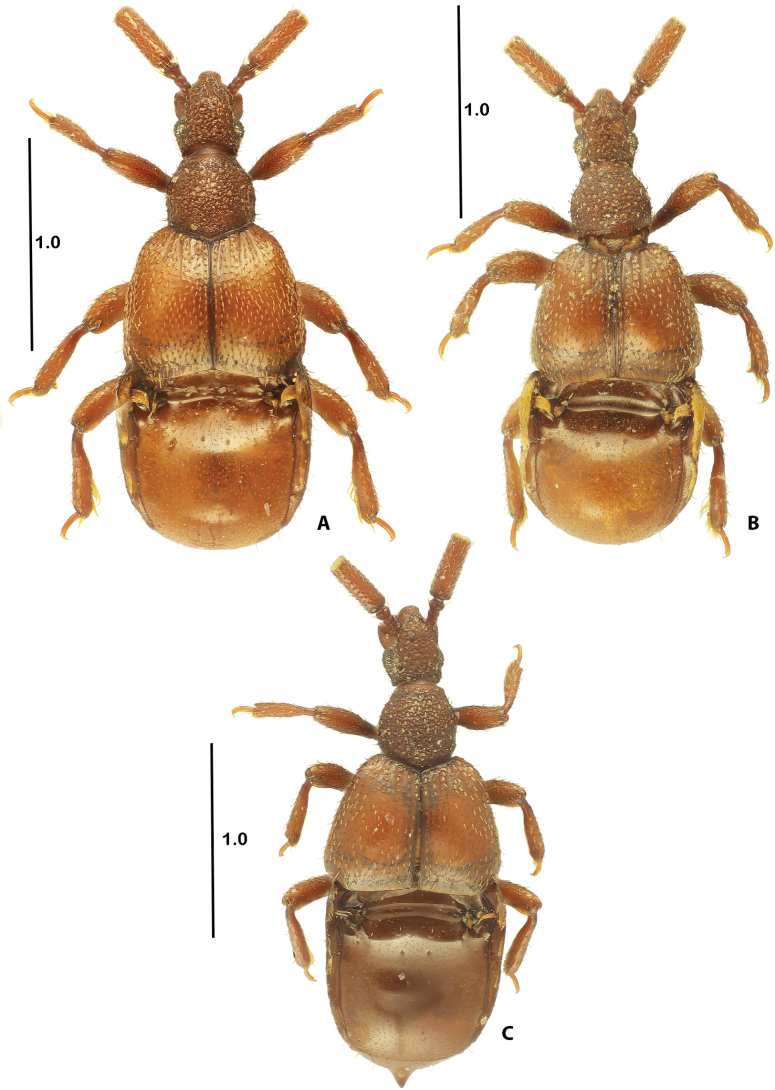
Dorsal habitus **A***Corynotopsisomanicus* sp. nov., holotype male **B***Corynotopsisscotti*, male **C***Corynotopsis* sp., female. Scale bars: 1.0 mm.

Body (Fig. 1A) length 2.20–2.30 mm, maximum width of elytra about 0.80–0.85 mm; reddish-brown, elytra slightly lighter, head and pronotum with rugose structure, elytra shiny with sparse short setae, abdomen shiny, glabrous.

Head elongate, about 1.20–1.25 times as long as wide; lacking frontal and vertexal foveae; frontal lateral margins parallel, rostrum narrow, expanded anteriad; clypeus massive, well-visible on sides as well as in front of rostrum; eyes prominent; temples slightly longer than length of eyes and slightly convergent posteriad. Disc of venter part of head smooth, sides with rugose surface, with separated posterior tentorial pits; well-separated gular plate shagreened.

Antenna hexamerous, about 0.5 mm long, slightly longer than head; scape minuscule, completely hidden in antennal cavity; antennomere 2 larger than scape and antennomere 3, slightly expanded distad, partly hidden in antennal cavity; antennomere 3 and 4 subequal, about as long as 2; 5 about as long as wide, 1.5 times as long as 4, strongly expanded distad; terminal antennomere longest, cylindrical, about five times as long as 5 and about three times as long as wide.

Pronotum 1.08–1.13 times as wide as long, about as long as head, widest before midlength, strongly convergent anteriad, slightly convergent posteriad; posterior margin triangular; lacking antebasal median foveae, with weakly-defined lateral fovea; lacking sulci.

Elytra 1.40–1.45 times as wide as long, 1.50–1.60 times as long as pronotum; lacking basal foveae, with short discal and sutural striae, almost reaching anterior third of elytral length; lateral posterior margin with weak, short trichome, sutural posterior margin with one long setae.

Mesoventrite shorter than metaventrite; mesocoxae narrowly separated by confluent posterior mesoventral and anterior metaventral process, isthmus about 0.25 diameter of mesocoxa, mesoventrite with median carina; metaventral disc with short setae, lacking trichome-like macrosetae, elevated, with medium, short, acute spine, in posterior third with large impression; posterior metaventral process wide, with slightly concave margin.

Abdomen long, 1.50–1.60 times as long as and 1.08–1.12 times as wide as elytra; basal basin of composite tergite transverse, almost entire but with two small protrusions forming two, small lateral lodges, basal basin long, in middle its posterior margin reaching half of length of composite tergite length; with lateral trichome born at foot of edge of paratergite I and directed mesad; with three, almost confluent paratergites, paratergite I and II with weakly-defined trichome on upper edge. First visible sternite (III) about half as long as second (IV), third and fourth (V–VI) about as long as first (III), fifth (VII) longer than fourth VI, sternites (IV–VII) with median impression.

Legs stout, all tibiae distally expanded; all femora and tibiae with interlocking ridges; mesofemur with basal thorn; mesotibia with predistal spine.

**Figure 2.**
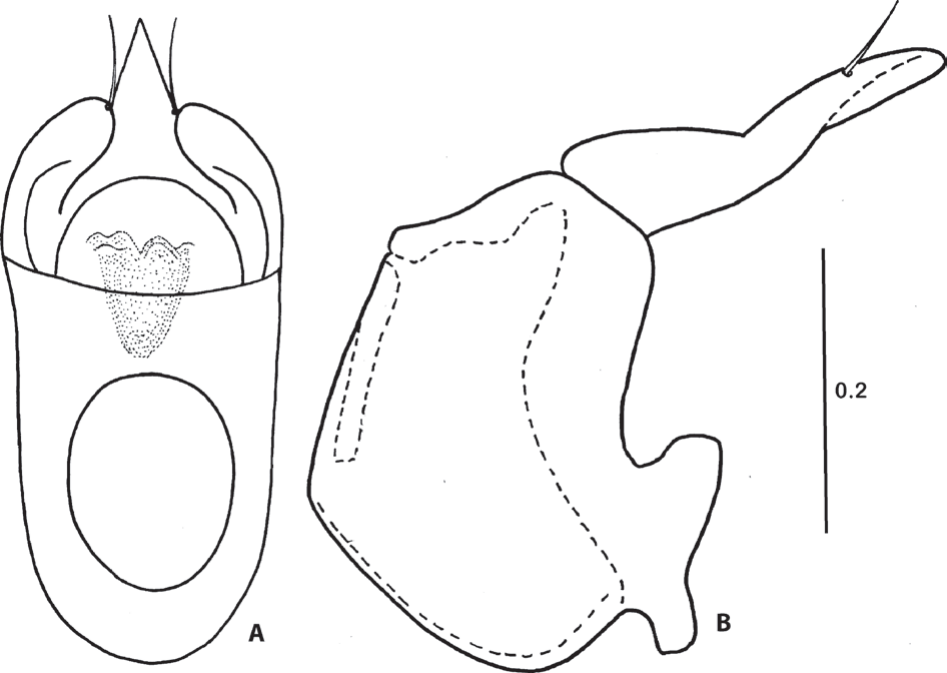
Aedeagus of *Corynotopsisomanicus* sp. nov., holotype male **A** dorsal view **B** lateral view. Scale bar: 0.2 mm.

Aedeagus (Fig. 2A, B) about 0.53 mm long, about 2.20 times as long as wide, basal capsule about 1.25 times as long as apical lobe; maximum width in distal third; apex of aedeagus sharply pointed, with pair of long setae; dorsal circular diaphragm well-defined.

#### Sexual dimorphism.

Females with all legs simple, lacking thorn or spines.

#### Natural history.

All specimens were collected at a UV light placed in a semi­dry habitat on the edge of a sandy wadi with *Acacia*, and in an open area with a small stream in the Baobab Forest (Fig. 5A, B); one specimen was beaten from branches of bushes at night. The host ant is unknown.

#### Etymology.

Locotypic, referring to the country of its type locality, Oman.

#### Distribution.

Oman (Dhofar Province).

#### Remarks.

*Corynotopsisomanicus* sp. nov. is very similar in external morphology to its congener *C.scotti* from which it can be distinguished only by: the different proportion of antennomeres 3 and 4; antennomere 3 and 4 subequal in length (antennomere 3 about 1.3 times longer than 4 in *C.scotti*); and by the different structure of the aedeagus with the distal projection of the median lobe triangular and wide at the base in ventral view, evenly narrowing to the apex (highly variable but much narrower at base and converging somewhat unevenly to apex in *C.scotti*).

### ﻿ Corynotopsis scotti

Taxon classificationAnimaliaColeopteraStaphylinidae

Jeannel, 1951

A320BC90-6C74-5B82-8779-0F7D325A97B6

**Figure 3.**
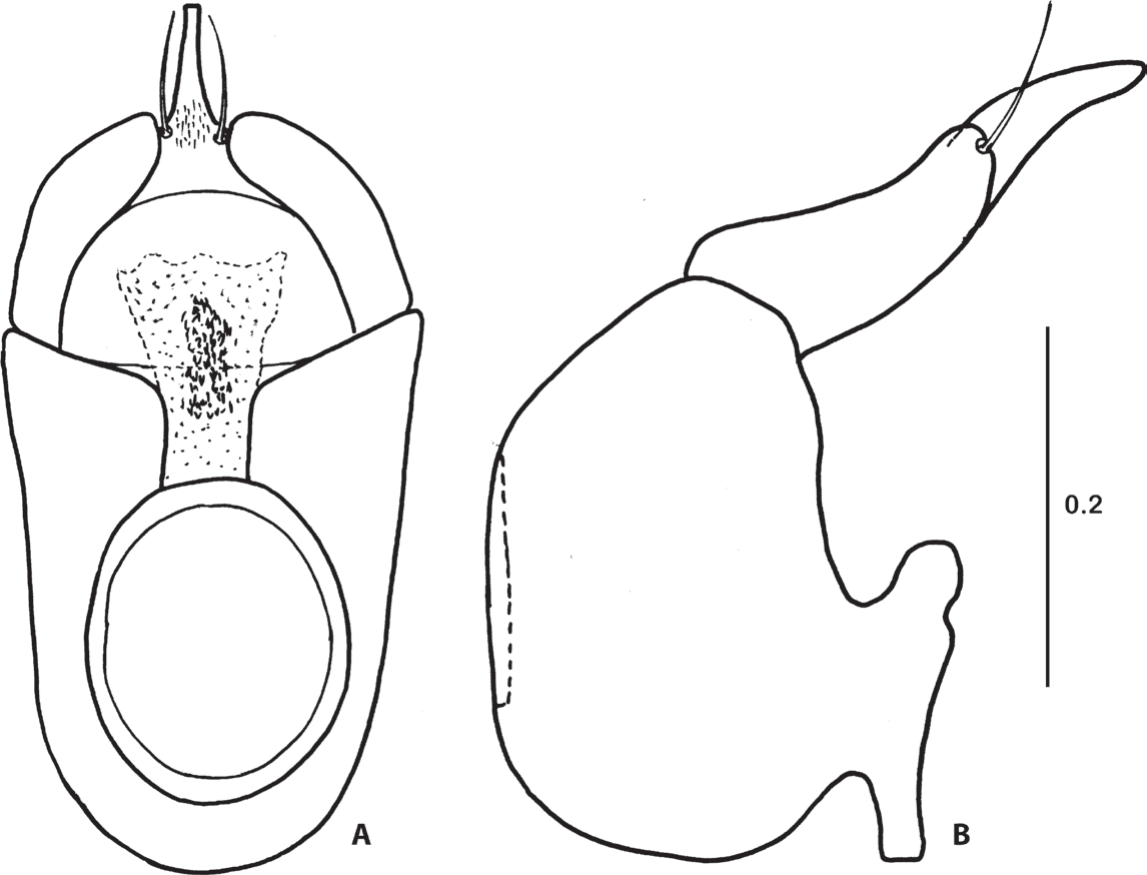
Aedeagus of *Corynotopsisscotti***A** dorsal view **B** lateral view. Scale bar: 0.2 mm.

**Figure 4.**
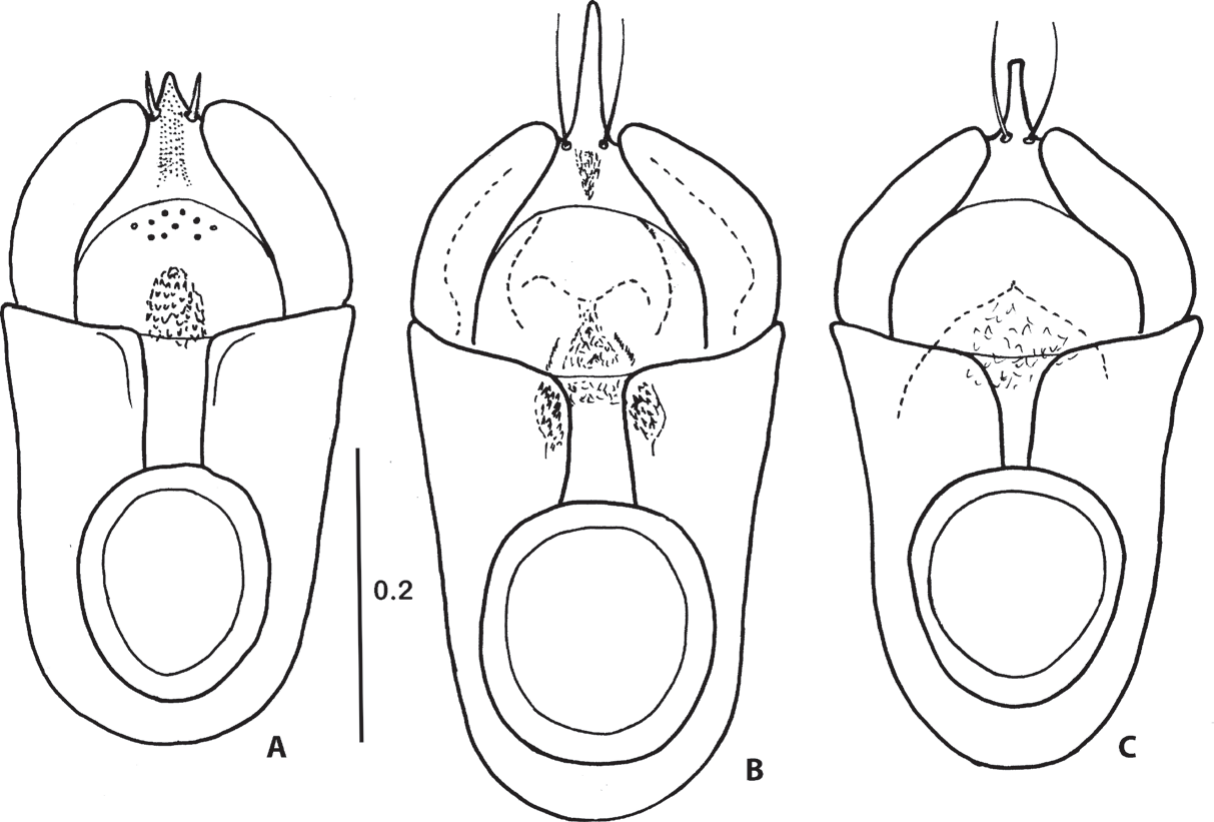
Variability of aedeagus of *Corynotopsisscotti*, dorsal view. Scale bar: 0.2 mm.

Figs 1B , 3A, B , 4A–C

**Figure 5.**
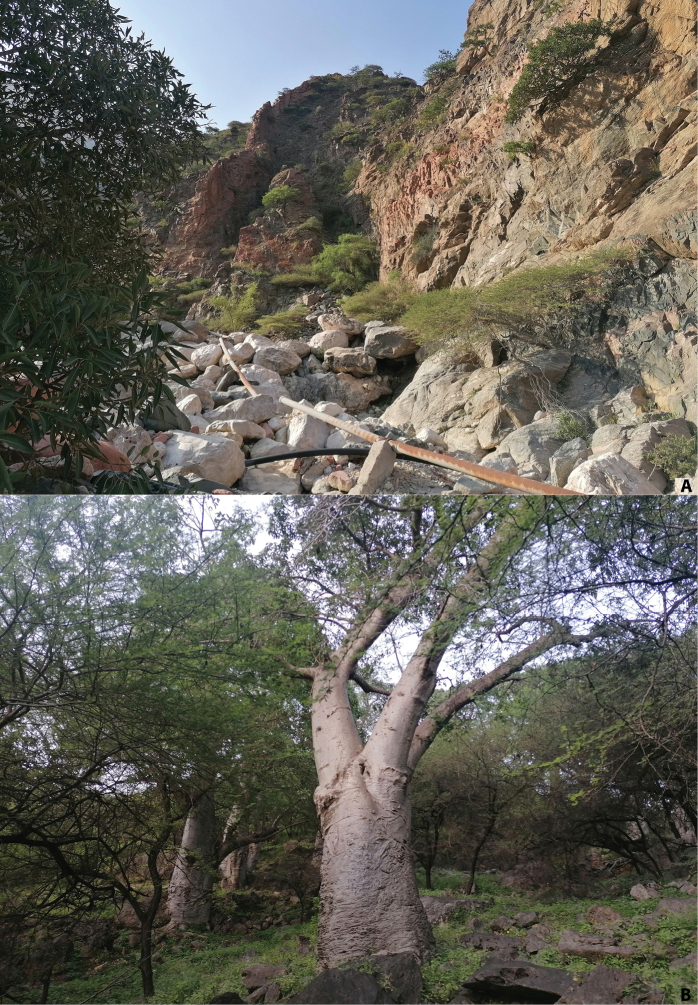
Type locality of *Corynotopsisomanicus* sp. nov.

**Figure 6.**
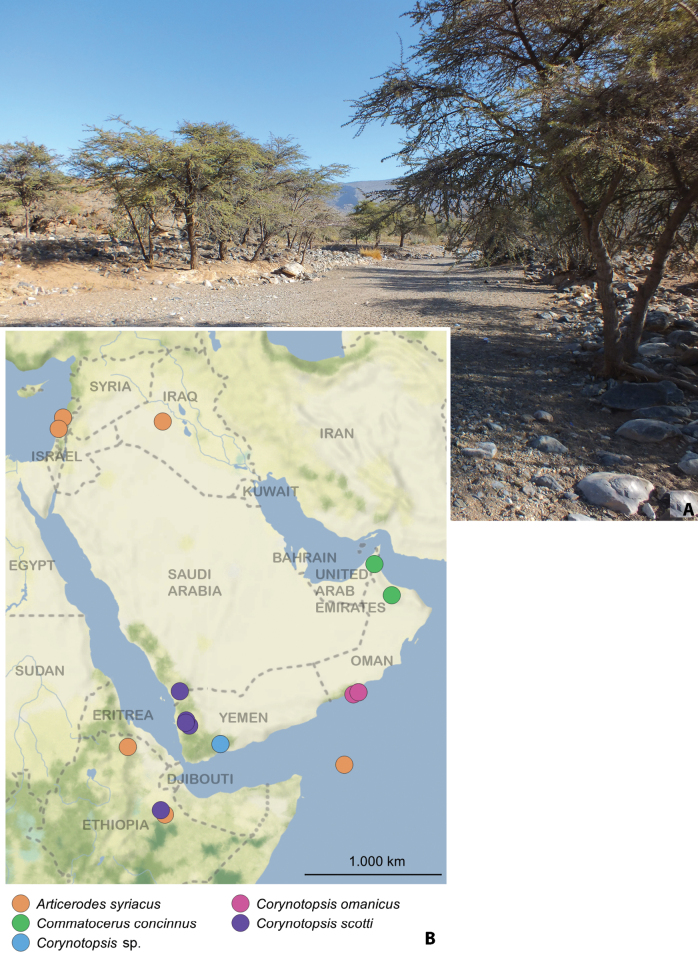
**A** Oman, Ad Dakhilyiah Gov., near Subayb wadi, locality with the occurrence of *Commatocerusconcinnus***B** distribution map of all Clavigeritae species known from the Arabian Peninsula.

Corynotopsis scotti Jeannel, 1951: 229, fig. 2 (habitus). Type locality: Ethiopia, Province of Harrar, Dire Dawa [Diré-Daoua], 9°36'3.15"N, 41°51'0.51"E, 2300 m.
Corynotopsis scotti Jeannel: Jeannel 1959: 695 (in key), 704 (distribution), fig. 257 (habitus); Coulon 1982: 67 (redescription), figs 5–6 (aedeagus).

#### Material examined.

1 ♂, **Yemen**: two labels “S Yemen, 27–28.III.2007 / 20 km W Lawdar / 13°53'N 45°48'E / ca 1100 m, David Král lgt.” [white, printed], ”*CORYNOTOPSIS* / *scotti* Jeannel / P. Hlaváč det., 2023” [white, printed] (NMPC). 2 ♂, 1 ♀: one label “YEMEN, 1101 m, 20 km / W Lawdar, 26–27.III.2007 / N13°53’ E45°48’ / P. Kabátek lgt.“ [white, printed] (MHNG, PCPH). 1 ♂: one label “YEMEN: Al Lahima / (6105) in Malaise trap / 24.VII-17.IX.2001 / leg. A. van Harten“ [white, printed] (MHNG). 1 ♂: one label “W YEMEN, Wadi Surdud / (Sari’) W San’a; N15°15’ / E43°30’, 627 m, 2.XI.2005 / leg. P. Kabátek lgt.“ [white, printed] (MHNG). 1 ♀: one label “YEMEN: 12 km NW of / (5986) Manakátekhah / in Malaise trap / 03.VII-21.VIII.2001 / leg. A. van Harten“ [white, printed] (MHNG);

#### Remarks.

*Corynotopsisscotti* was described by Jeannel (1951) based on a single male from Ethiopia (Prov. de Harrar: Diré-Daoua [Dire Dawa], 2300 m). Later, the holotype was studied, the species was redescribed and the illustration of the aedeagus was provided by Coulon (1982). Besuchet (1999) provided a record of one female from Saudi Arabia. The species is also mentioned from Yemen in the last edition of the Palaearctic Coleoptera (Schülke and Smetana 2015), but the source of this record is unknown to us. New record for Yemen.

Species of the genus *Corynotopsis* are very similar concerning the external morphology. The only external difference between *C.scotti* and *C.omanicus* is a slightly different proportion of antennomeres 3 and 4 (see remarks for *C.omanicus*). All specimens from Yemen, assigned here to *C.scotti*, have quite different shapes of the distal part of the medial lobe of the aedeagus in ventral view (Figs 3A, 4A–C). The main differences are in the length and shape of the distal projection of the median lobe and its size relative to the pair of distal setae. To solve the question of whether this is just intraspecific variability or a complex of more closely related species, more material will be needed.

#### Host ant.

Unknown.

#### Distribution.

Ethiopia, Saudi Arabia, Yemen.

### ﻿ Corynotopsis

Taxon classificationAnimaliaColeopteraStaphylinidae

sp.

7B5A1179-F092-54AE-8BB7-3D755DA864C0

Fig. 1C

#### Material studied.

1 ♀: **Yemen**: one label “YEMEN, 1101 m, 20 km / W Lawdar, 26–27.III.2007 / N13°53’ E45°48’ / P. Kabátek lgt.“ [white, printed] (MHNG).

#### Remarks.

This single female certainly represents a new, undescribed species. It is readily separated from all other females of *Corynotopsis* by: 1) the larger body, length 2.43 mm, maximal width 0.94 mm; 2) the pronotum slightly longer than wide; 3) the pair of paratergal trichomes, fine, formed by one to two macrosetae; and mainly; and 4) by having the sharp, pointed projection on the end of the abdomen. Due to the absence of a male and taking into account the complicated taxonomic situation in *C.scotti* (see Remarks for that species), we decided not to formally describe this species.

### ﻿ Articerodes syriacus

Taxon classificationAnimaliaColeopteraStaphylinidae

Saulcy, 1865

A0DB8931-96DC-5317-8A97-9FFDFF6C592D

Articerus syriacus Saulcy, 1865: 25. Type locality: Saïda [Sidón], en Syrie; types: HT, probably ♂.
Articerus ponticus Sharp, 1878: 62. Type locality: Mésopotamie; types: unknown, synonymy in Raffray 1890: 167.
Commatocerus bucharicus Reitter, 1900: 50. Type locality: Transcaspien, Buchara, Karatak; types: more ST, synonymy in Winkler 1925: 470.
Commatocerus sbg. Articerussubnitidus Pic, 1903: 145. Type locality: Crete; types: more ST, synonymy in Besuchet 1999: 63 (as C.subnitidius [sic]).
Articerus spriacus Saulcy: King 1869: 57. [error]

#### Host ant.

Lasius (Lasius) niger (Linnaeus, 1758); *Lepisiotacapensis* Mayr, 1862; *L.canescens* Emery, 1897 and *L.spinisquama* (Kuznetsov-Ugamsky, 1929).

#### Distribution.

Greece (Crete), Turkey, Lebanon, Syria, Israel, Iraq, Iran, Yemen (Socotra I..), Ethiopia, Tajikistan, Uzbekistan.

#### Remarks.

The genus *Articerodes*Raffray 1890, comprising 11 species, is distributed in a large area from the Republic of South Africa, through the Democratic Republic of Congo, Ethiopia, Middle East, Central Asia, southern India, Indochina, and north to Japan. Such a large distribution is relatively unusual for genera of Clavigeritae and it is possible that not all species will remain in the genus. The type species of the genus, *Articerodessyriacus* Saulcy, 1865, is also unusually widespread. We tried to find all localities from where the species was recorded and they are listed here. The only locality listed in the last edition of the Palaearctic catalogue (Schülke and Smetana 2015) which remains unknown to us is Iran.

#### Literature records.

**Ethiopia**, Mekallé in Enderta and Harrar; **Greece**, Crete (type locality of *C.subnitidus*); **Iraq**, as Mesopotamia, but more precise information on the locality is unknown (type locality of *C.ponticus*); **Israel**, St.-Jean-d’Acre [Akko]; **Lebanon**, Sidón (type locality *C.syriacus*); **Uzbekistan**, Buchara, Karakat (type locality of *C.bucharicus*); **Yemen**, island Socotra, Al Haghier Mts., wadi Madar, 1180–1230 m 12°33.2'N, 54°00.4'E.

#### New records.

**Turkey**, 1 ♀: one label “N36°50’ E028°42’ / T Umg. Mugla / Köycegiz; Auenwald / 29.4.2001 / Meybohm/Brachat“ (PCPH). 1 ♂: one label “Türkei / Südküste / zw. Antalya u. Alanya / 22.5–3.6.1983 / leg. V u. C. Brachat“ (PCPH). **Tadjikistan**, 1 ex, one label “Mts. Karategthin [Rasht Valley], Baldshuan, 924 m“ (PCPH).

### ﻿ Commatocerus concinnus

Taxon classificationAnimaliaColeopteraStaphylinidae

Besuchet & Cuccodoro, 2011

1FC3B260-550E-555C-AAA0-6144B160A53E

Commatocerus concinnus Besuchet & Cuccodoro, 2011: 165, plate 14 (habitus), figs 22–24 (aedeagus); type locality: United Arab Emirates, Ras al Khaimah, Wadi Shawkah, 25°6'15.30"N, 56°2'47.55"E.

#### Material examined.

1 ♂, 1 ♀, **Oman**: two labels “Oman, 28–29.3.2019, Ad / Dakhilyiah Gov., near Subayb / 23°14'7.966"N, 57°8'57.977966"E, / wadi, 1442 m, Lgt. T. Kopecký“ [white, printed], “*COMMATOCERUS* / *concinnus* Bes & Cucc. / P. Hlaváč det., 2023“ [white, printed] (PCTK, PCPH). New record for Oman.

#### Natural history.

The two specimens were collected from two different ant nests under stones. One with an unknown species of *Lasius* Fabricius, 1804 and the other with *Lepisiota* (cf.) *spinisquama* (Kuznetsov-Ugamsky, 1929), M. Sharaf det., 2023, collected in the evening on an open, dry area in the mountains (Fig. 6A).

#### Remarks.

*Commatocerusconcinnus* was described based on one male from United Arab Emirates, Ras al Khaimah, Wadi Shawkah, 25°6'15.30"N, 56°2'47.55"E, collected by a water-trap. The species is readily separated from its congeners by having the terminal antennomeres cylindrical, instead of distally expanded.

The generic name *Commatocerus* has been subjected to much instability. It was established by Raffray (1882: 1) for *Commatoceruselegantulus* Raffray. In the same year, Reitter (1882) synonymized it with the genus *Fustiger* LeConte, 1866 under the name *Comatocerus* [sic] (Reitter 1882: 200). Jeannel (1949: 37) redescribed the genus and resurrected its generic status. He also pointed out that the American and Old World species of the genus *Fustiger* cannot be congeneric and should be placed in separate genera. Later, Jeannel (1954: 152) discussed the status of similar genera *Commatocerus*, *Fustigerinus* Wasmann, 1912 and its actual synonym *Neocommatocerus* Jeannel, 1949. Célis (1975: 441) confirmed the generic status of *Commatocerus*. Besuchet (1977: 261) again synonymized it with *Fustiger* and provided a key to species of India. When describing *Commatocerusconcinnus* (Besuchet and Cuccodoro 2011: 164), the authors discussed and supported the validity of the genus and they apparently overlooked the synonymy of Besuchet from 1977.

#### Species included.

*Commatocerusconcinnus* Besuchet & Cuccodoro, 2011, *C.elegantulus* Raffray, 1882, *C.leleupi* Jeannel, 1953 and *C.turkmenicus* Kryzhanovski, 1957.

## Supplementary Material

XML Treatment for Corynotopsis omanicus

XML Treatment for Corynotopsis scotti

XML Treatment for Corynotopsis

XML Treatment for Articerodes syriacus

XML Treatment for Commatocerus concinnus

## ﻿Additional information

### Author ORCIDs

Dominik Stočes https://orcid.org/0000-0002-1867-5327

### Data availability

All of the data that support the findings of this study are available in the main text.
